# Oral health and mortality risk in the institutionalised elderly

**DOI:** 10.4317/medoral.17632

**Published:** 2012-02-09

**Authors:** Dairo J. Marín-Zuluaga, Leiv Sandvik, José A. Gil-Montoya, Tiril Willumsen

**Affiliations:** 1 The Gedorontology Group, Oral Health Department, Faculty of Dentistry, Universidad Nacional de Colombia, Bogotá, Colombia; 2 Cariology and Gerodontology Department, Faculty of Dentistry, University of Oslo, Oslo, Norway; 3 Department of Special Care in Dentistry and Gerodontology, Faculty of Dentistry, University of Granada, Spain

## Abstract

Objective: Examining oral health and oral hygiene as predictors of subsequent one-year survival in the institutionalized elderly. Design: It was hypothesized that oral health would be related to mortality in an institutionalized geriatric population. A 12-month prospective study of 292 elderly residing in nine geriatric institutions in Granada, Spain, was thus carried out to evaluate the association between oral health and mortality. Independent samples, T-test, chi-square test and Cox regression analysis were used to analyze the data. Sixty-three participants died during the 12-month follow-up. Results: Mortality was increased in denture users (RR = 2.18, p= 0.007) and in people suffering severe cognitive impairment (RR = 2. 24, p= 0.003). One-year mortality was 50% in participants having both these characteristics. Conclusions: Oral hygiene was not significantly associated with mortality. Cognitive impairment and wearing dentures increased the risk of death. One-year mortality was 50% in cognitively impaired residents wearing dentures as opposed to 10% in patients without dentures and cognitive impairment.

** Key words:**Oral health, mortality risk, institutionalised elderly.

## Introduction

Average life-span has been increasing all around the world and also in the elderly population. Oral health is related to general health, cognitive status and quality of life (1,2); these aspects have been found to be predictors of late-life survival (3). The elderly are expected to preserve most of their teeth in the future, particularly in developed countries, but current cohorts of elderly have lost a lot of teeth throughout their lives. Dental status results from accumulated oral infections (among other factors); in the elderly it reflects lifelong experiences of caries and periodontal disease as well as socioeconomic status, life-style and attitudes towards dental care (4). Loss of teeth has been found to affect masticatory ability (5), to influences the selection of food and nutritional status (6) and to have a negative impact on oral-related quality of life (QoL) (7-9).

Several studies have addressed whether dental status is associated with mortality. Heitmann et al., (10) concluded that tooth loss indicates a high risk for cardiovascular disease and stroke. Poor dentition, especially edentulousness, has been associated with deterioration in the systemic health and higher mortality of the aged (3,11-12). However, the age-range has been broad in many studies, but relatively few have been limited to an 80+ population. Hamalainen et al., (13) found the hazard ratio for death associated with a decrease of one missing tooth was 1.026 (p<0.05) in a 10-year cohort study. Ansai et al., (14) found tooth-loss to be a significant predictor of mortality, even when controlling for socio-economic status.

Poor oral hygiene may be considered a measure of current oral infection level. Proper oral hygiene has been found to be important in preventing death from aspiration pneumonia in nursing homes (15). Sjøgren et al., (16) concluded that around one in 10 cases of death from pneumonia in elderly nursing-home residents might have been prevented by improving oral hygiene.

It was thus hypothesized that oral health would affect mortality in an institutionalized geriatric population. The present study was aimed at examining oral health and oral hygiene as predictors of subsequent one-year survival in the institutionalized elderly.

## Material and Methods

This study forms part of a longitudinal study (the main study) on a population consisting of institutionalized people aged 52–102 living in the Province of Granada, Spain. Data was collected from April 2009 to September 2010. The main study’s inclusion criteria were to have at least three natural teeth and/or to wear dentures. 369 residents were examined at baseline. During the 12-month follow-up period 102 participants were retired from the study, 66 because they died and 36 because of other causes.

The participants were interviewed and given a dental examination at their institutions in a room guaranteeing acceptable privacy. Head nurses, physicians and residents’ relatives were asked to provide information where necessary because of cognitive impairment. A headlamp and a mouth mirror were used during oral examination. An experienced dentist in Gerontology (first author) collected all data.

The present paper includes all participants older than 75 from the main study. This left 292 participants; 63 died within the first year and 229 survived. The participants who died were categorized into: (A) died within the first three months after examination, (B) died within the first six months after examination, (C) died within the first nine months after examination and (D) died within the first twelve months after examination.

## Measurement

-Background variables

Age and gender was recorded, as was *educational level* (low = no studies or primary school, medium = high school and high = technical or university studies).

-Nursing and general medical variables

Independence for dressing and washing and independence for oral hygiene were categorized into three levels (independent, some help needed and dependent). Their medical histories were checked for obtaining data on *entry to institutions and the medicines being used*. A doctor estimated the *number of pathologies* from the medicines each participant was using.

Cognitive state was established by using the Pfeiffer test (17) (a 10-question screening instrument covering orientation, recent memory, retrospective memory, attention and calculus). Final scores range from 4 (normal), 3 (mild cognitive impairment), 2 (moderate cognitive impairment) to 1 (severe cognitive impairment). Participants unable to answer because they obviously had severe cognitive impairment or dementia directly scored 1.

-Oral health variables

Use of dental services was evaluated by asking about regular oral check-up frequency (each 6-12 months, only if needed) and time since the last dental visit (6-12 months, 1-2 year, >2 years).

Dental status was recorded as being the number of visible natural teeth, occluding pairs (natural teeth having a natural or prosthetic antagonist), retained roots, and dental caries (visually examined and recorded by tooth as being crown caries or root caries; this was recorded as root caries when a lesion affected both crown and root).

Oral hygiene was measured using Sunstar dental disclosing tablets (G-U-M/MD Americas Inc. Chicago, IL 60630 USA) for disclosing dental and denture plaque. Residents having remaining natural teeth were asked to chew one tablet for around 30 seconds. Mouths were then rinsed with water. The simplified oral hygiene index (OHI-S) (18) was recorded for all residents who had at least two of the teeth required by this index. The O’Leary Index (overall percentage of plaque) (19) was used for all who had at least one natural remaining tooth. The denture hygiene index (DHI) (20) was recorded by dissolving five dental disclosing tablets in 50cc of water into which the dentures (previously rinsed with water) were placed for 30 seconds and then rinsed with running water. Denture cleanness was evaluated as being excellent (none or only a few spots of plaque), fair (more extended plaque, less than half the denture base covered by plaque) and poor (more than half the denture base covered by plaque).

Dental status and the presence of dentures made it impossible to use the same oral hygiene index for all participants. A new global oral hygiene variable was calculated from the following criteria to include all participants in the same analysis: first priority included the OHI-S category, the second priority (if not enough teeth present for OHI-S) the DHI value and third priority (if neither OHI-S nor DHI were available) the percentage of plaque. The global oral hygiene score was categorized into the following criteria: 1= excellent (OHI-S score below 0.6 or DHI score = 1 or less than 50% overall plaque score), 2 = acceptable (acceptable OHI-S score (0.7-1.6) or DHI score = 2 or 50%-80% overall plaque score) and 3 = unacceptable (unacceptable OHI-S score (above 1.6) or DHI score = 3 or >80% overall plaque score).

Survival: participants who died were recorded at 3, 6, 9 and 12 months.

-Statistical analysis

The Statistical Package for Social Sciences (Version 15.0) (SPSS Inc., Chicago, IL, USA) was used for data analysis. All variables regarding group differences were tested using independent T-tests for numerical data and the Mann-Whitney test for skewed numerical or categorical data. Kaplan Meier plots with log-rank test were used for identifying factors significantly associated with survival (bi-variate analysis). Cox regression analysis was used for multivariate analysis. Inclusion criteria for Cox regression analysis were (1) p<0.20 Kaplan Meier, (2) VIF <2.5 collinearity. A 5% significance level was used throughout.

## Results

Most of the 292 participants were women (228, 78.2%). Their ages ranged from 75 to 102 (mean = 85.3 years). 74.5% of the participants had a low educational level. About a quarter of the residents (81, 27.7%) were dependent on help for dressing and washing, and 76 (26%) depended on assistance for tooth cleaning.

The number of medicines varied from 0 (3.4%) to 20 (0.3%) with a mean of 7.3 (SD 3.8). Number of pathologies varied between 0 (3.4%) and 7 (1.4%) (mean 3.4, SD 1.4). The most usual pathological diagnoses were hypertension (61.6%), gastritis (50.3 %), depression (26.0%), psychosomatic pain (16.1%), cardiac pathology (15.4 %), insomnia (13.7%), constipation (13.4%), hyper-cholesterolemia (11.6%), psychosis (9.2%), eye-related diseases (7.2%) and respiratory system diseases (6.8%).

According to the Pfeiffer test, 130 (44.5%) participants had normal cognitive function, 58 (19.9%) had mild cognitive impairment, 49 (16.8%) had moderate cognitive impairment and 55 (18.8%) had severe cognitive impairment. There was no statistical significant difference between men / women as regards cognitive impairment (p=0.08) or being dentate / edentulous (p=0.6).

Most participants made use of dental services only when needed (81.5%) and 59.2% had not been to the dentist for more than two years. Significantly more dentate participants regularly went to a dentist than edentulous ones.

-Oral status 

Most residents had remaining teeth. The mean number of teeth was 8.2 (range 0-30), 95 (32.5%) were edentulous, 44 (15.1%) had more than 20 teeth and 175 (59.9%) wore dentures. Among participants having remaining teeth, the mean number of decayed teeth was 1.1 (range 0-10). There was a significant difference between people who died and survived as regards having less than seven remaining teeth (p=0.04).  Table 1 shows background and oral health variables among survivors and participants who died.

**Table 1:**
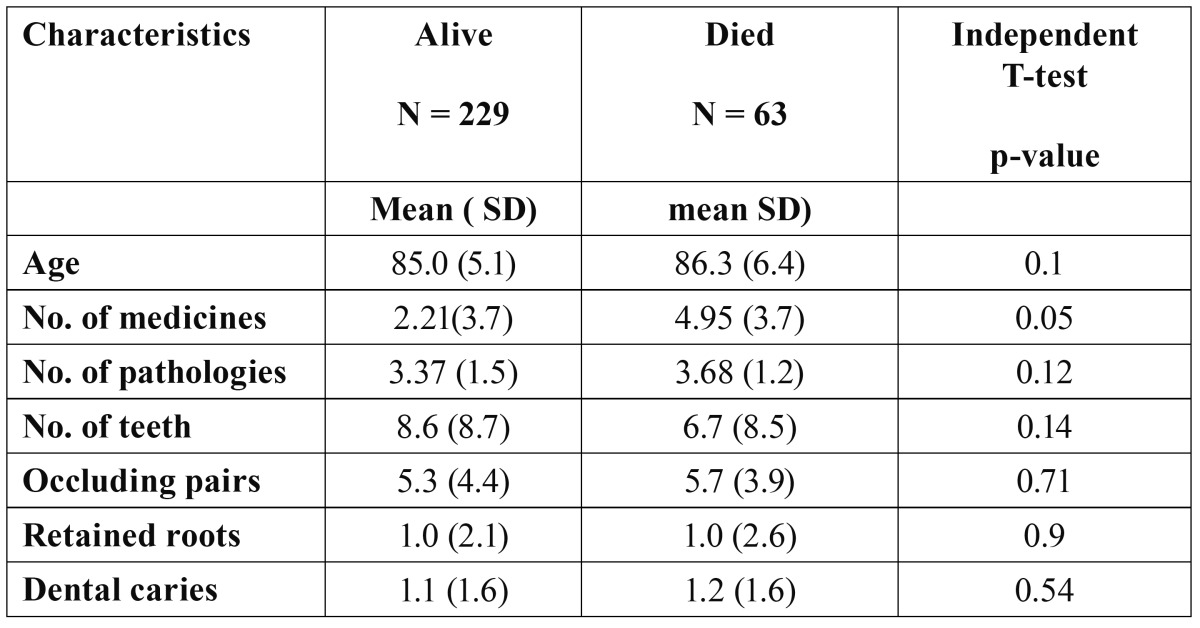
Background variables for those who survived and those who died within the first 12 months after examination.

-Oral hygiene

Only 37 participants (12.7%) had excellent oral hygiene, 78 (26.7%) were rated acceptable but most (177, 60.6%) had unacceptable oral hygiene. There were no significant differences between men/women regarding the use of medications or having more than 10 teeth. Significantly more residents suffering severe cognitive impairment had unacceptable oral hygiene (p=0.001).

All 12 factors fulfilled collinearity inclusion criteria (p<0.2) ( Table 2). All these factors were thus simultaneously included in the Cox regression analysis. The following two factors remained after stepwise backward variable selection until all remaining factors became statistically significant (p<0.05): severe cognitive impairment and denture use. Severe cognitive impairment increased mortality by 120% (HR=2.24, p=0.003) and denture use increased mortality by 120% (HR=2.18, p=0.007).

**Table 2:**
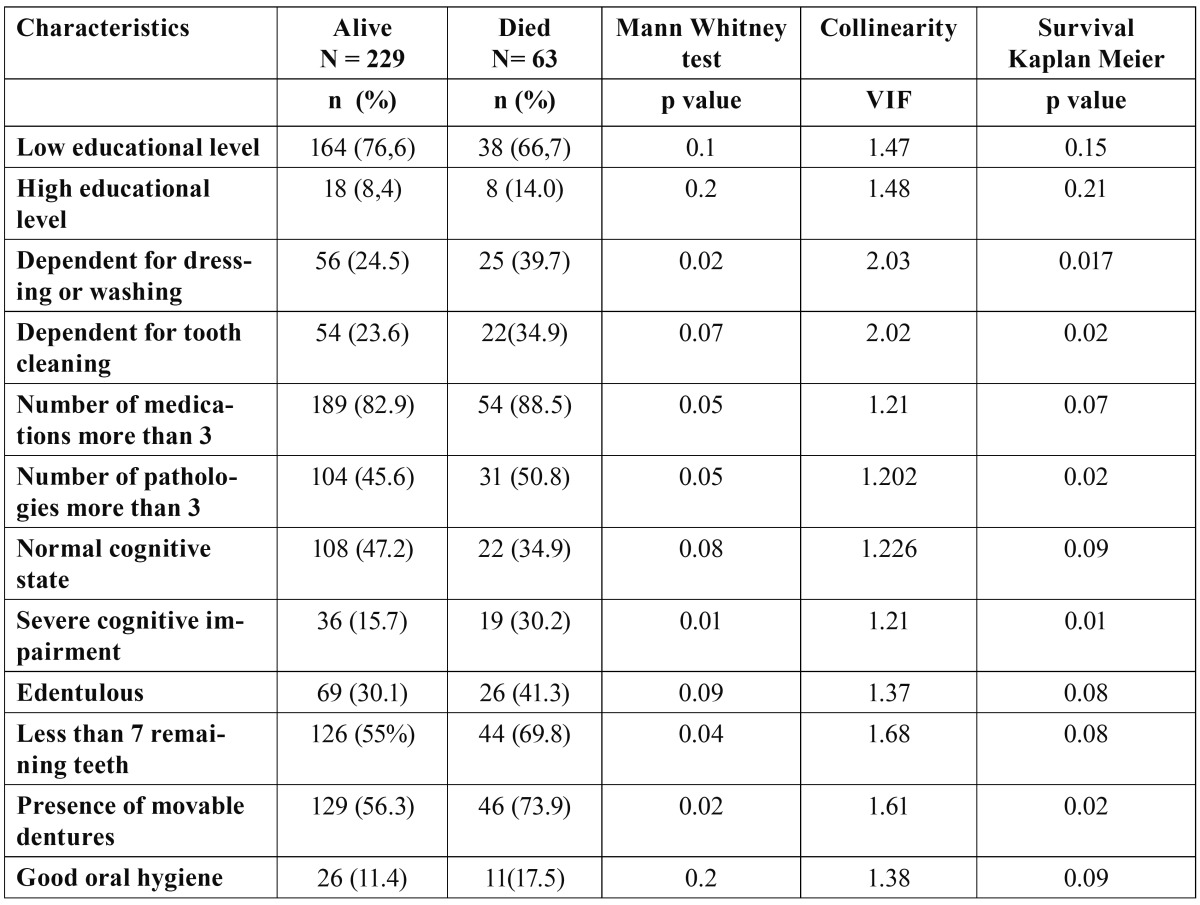
Variables which met inclusion criteria (p<0.2) for Cox regression analysis.

**Figure 1:**
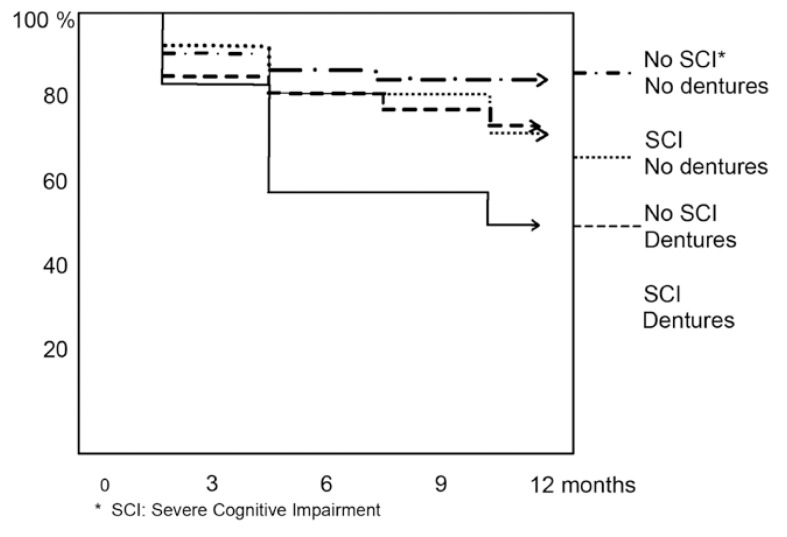
Survival rate.

The participants were categorized into 4 groups to further illustrate how these two factors were associated with mortality: (1) no denture and no severe cognitive impairment (n=86), (2) no denture and severe cognitive impairment (n=151), (3) denture and no severe cognitive impairment (n=31) and (4) denture and severe cognitive impairment (n=24). These four groups’ Kaplan Meier regression curves are shown in (Fig. 1). 10% of participants having no denture and no severe cognitive impairment died during one year as opposed to 50% of participants wearing dentures and suffering severe cognitive impairment.

## Discussion

This study’s main findings were that wearing dentures increased mortality even when controlled for age, severe cognitive impairment, educational level, needing help for dressing or washing and needing help for tooth cleaning. Thus, having only natural teeth and no dentures appears to increase one-year survival. Being cognitively impaired also increased the risk of death. One-year mortality was 50% when wearing dentures and also being cognitively impaired. Oral hygiene had no impact on survival rate.

Aging has been considered the most important risk factor for physical and mental disorders and death (21). However, it was not significantly difference at baseline between the age of those who died or survived in our study on a population aged 75+ and the mortality risk of denture users was significantly higher, even after being controlled for age. Our results support earlier studies that have reported denture use as a mortality risk. Fukai et al., (22) found that wearing dentures was one of the factors associated with mortality in a 15-year follow-up study on a sample of people aged 40+. Furthermore, Shimazaki et al., have found that people having the worst dentition status (edentulous subjects without dentures) suffered significantly increased mortality, independent of physical-mental health status at baseline and concluded that maintaining more functional occlusion (with natural teeth or dentures) may lead to longer life expectancy (12).

Being severely cognitive impaired in our study increased the risk of death by 120%. Thorstensson et al., reported similar findings in a 10-year study on Swedish octogenarian twins. They found cognitive status to be the overall survival predictor, independently of age or gender (3). The present study found that the risk of dying within a year was substantial when joining the two main explanatory variables (wearing dentures and having severe cognitive impairment).

It could be speculated that high mortality rate among denture wearers suffering severe cognitive impairment could represent an increased masticatory disability. Chewing ability, when using dentures, depends on both muscular strength and neuromuscular control. Severe cognitive impairment could alter neuromuscular control, thereby affecting chewing performance. It is a common clinical observation that dentures (especially lower full dentures) are often left unused in demented people and their chewing ability consequently becomes worse.

Tooth loss also affects masticatory functioning (23) and altered chewing ability is associated with a diet low in ingredients like plant food (24); low plant food intake is associated with worse cognitive function (25). Patients’ health may thus be lead into a vicious circle involving decreased general health, lower cognitive function and increased risk of death. Chewing ability has also been found to be associated with a greater risk of mortality in community-residing elderly people by Nakanishi et al., who evaluated self-assessed masticatory ability in dentate and denture users amongst community-residing elderly in a 9-year mortality cohort study (26).

Denture use results from loss of teeth, reflecting a cumulative experience of oral infections as caries and periodontal disease (27). Although the number of teeth, pathologies or medications were not found to be strong predictors of death in the regression analysis, there were significant differences in univariate analysis regarding these variables between survivors and participants who died. Significantly more people who survived had more than 7 teeth in our study, indicating that the number of teeth is an important factor for survival rate. This agreed with Hamalainen et al., who concluded that, the more teeth or filled teeth a subject had, the smaller their risk of death (13). Osterberg et al., also found that each remaining tooth at age 70 decreased 7-year mortality risk by 4% (28). Loss of teeth may be associated with other health risks such as smoking, diet and lifestyle (4), thereby reflecting a persons’ general health and mortality risk. It has also been associated with an increased risk of death, independently of health factors, socio-economic status and lifestyle (14,29).

Sjogren, in a systematic review of randomized controlled trials, concluded that mechanical oral hygiene has a preventative effect on mortality from pneumonia and that about one in 10 cases of death from pneumonia in elderly nursing home residents may be prevented by improving oral hygiene (16). Even if significantly more residents suffering from severe cognitive impairment had unacceptable oral hygiene in our sample, oral hygiene had no impact on survival rate. One explanation may be that no deaths were reported as being due to pneumonia. Even if not associated with survival rate, dental plaque is important as the main cause of dental caries and periodontal disease (i.e. the most prevalent oral diseases) as both cause loss of teeth (associated with decreased oral-related QoL (30) and increased risk of death) and periodontal disease has been reported as being associated with the risk of death among elderly people (25).

Our findings let us accept our working hypothesis and state that oral health increased mortality risk in our sample of the institutionalized elderly.

Some of the present study’s limitations need to be discussed. The sampling method was not random and only nine of the 54 geriatric institutions in Granada participated in the study (though they were considered to be representative of this population). A potential selection bias, although not clearly apparent, cannot thus be ignored. Data regarding mortality causes were not provided by most of the institutions that took part in this study, therefore it was not possible to control by this important variable. It was difficult to get information about the systemic diagnostics of the residents, and because of this a physician had to estimate the number and kind of pathologies from the medicines each participant was using. This in turn created some uncertainty about the pathologies each patient was suffering so we decided to exclude this variable from the analysis. Because of this results from the current study should be seen as a first look at this issue in the studied population, and as such should be interpreted with caution.

## Conclusion

Oral hygiene had no impact on survival rate. Cognitive impairment and use of dentures increased the risk of death. The risk of death within a year was 50% in cognitively-impaired residents wearing dentures.
